# Intraoperative push enteroscopy for treatment of occult small bowel bleed due to hemorrhagic bleed and tumor: a report of two cases

**DOI:** 10.1093/jscr/rjad271

**Published:** 2023-06-30

**Authors:** Maribona A Sofia, Philip Dwight, Shatawi Zaineb, Seaver Christopher

**Affiliations:** Herbert Wertheim College of Medicine, Florida International University, Miami, FL, USA; Herbert Wertheim College of Medicine, Florida International University, Miami, FL, USA; Division of General Surgery, Memorial Hospital West, Pembroke Pines, FL, USA; Division of General Surgery, Memorial Hospital West, Pembroke Pines, FL, USA

## Abstract

Small bowel bleeds, while uncommon, are often challenging with regard to diagnosis and therapeutic intervention. This is primarily due to their occult nature, the location offending lesions and limitations of current technology used to assess them. This review highlights two patients who presented with signs and symptoms of a small bowel bleed, where initial diagnostic workups were inconclusive, and intraoperative enteroscopy served a diagnostic and therapeutic role. We discuss the current literature on intraoperative endoscopy and propose an algorithm that introduces intraoperative enteroscopy earlier as a viable curative option, particularly in a rural setting. This case series proposes considerations for earlier introduction of intraoperative enteroscopy for diagnosis and treatment of small bowel bleeds.

## INTRODUCTION

Small intestinal bleeds are relatively uncommon, only accounting for 5–10% of gastrointestinal (GI) bleeding [1]. Such GI bleeds can be occult or overt. Occult bleeding refers to a positive fecal occult blood test in a patient with no visible evidence of blood loss, while overt bleeding describes bleeding that is visible to the patient or clinician in the form of melena, hematochezia or symptomatic iron deficiency anemia [1]. Due to the nature and locations of these lesions, most cannot be identified with standard endoscopic evaluation or imaging and require further workup with capsule endoscopy (CE), push enteroscopy (PE), device-assisted enteroscopy (DAE) radiologic evaluation and intraoperative enteroscopy (IOE).

IOE is considered an invasive procedure used to evaluate the small bowel during surgery, requiring an endoscopist and surgeon. Although it has a high diagnostic yield, IOE is associated with a morbidity and mortality (17 and 5%, respectively) [2]. These rates can be attributed to the acutely unstable status of patients who often undergo this procedure, rather than the procedure itself. The current role of IOE has been poorly studied alongside newer diagnostic techniques (DAE and CE), with only a few publications to date, namely articles by Voron *et al*. and Pat *et al.* Such studies have identified IOE as an important technique for the diagnosis and guidance of management in small bowel bleeding for patients where previous workup was inconclusive or could not be performed [2]. It is the most thorough way to inspect the small bowel for both serosal and mucosal lesions, and often detects new or additional lesions not previously identified preoperatively [3]. As such, it is imperative for surgeons to understand how to perform this procedure and their role in the management of distal small bowel bleeding. In this paper, we report two cases of obscure GI bleeding with very different clinical presentations, in which IOE was used to effectively treat the small bowel bleeding identified.

## CASE SERIES

**Figure 1:**
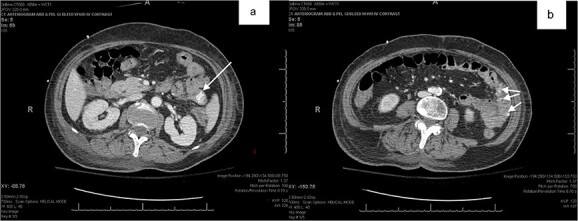
(**a**,**b**) abdominal CT with oral contrast identifying extravasation in the jejunal loop.

Patient 1 was a 70-year-old female with breast cancer (Grade 2 Intraductal Carcinoma) diagnosed in 2019, currently undergoing chemotherapy via a left chest port. She presented to the ER for a syncopal episode. She recently underwent port placement 2 weeks prior to presentation and received her first cycle of chemotherapy 5 days prior, which she did not tolerate well. She reported severe generalized weakness, joint pains and nausea since treatment, and one episode of emesis just before admission. Her hospital stay was complicated by declining renal function, melena and down trending hemoglobin, necessitating multiple transfusions of platelets and packed red blood cells (PRBCs). Esophagogastroduodenoscopy (EGD) showed an esophageal ulcer and gastroduodenitis, and colonoscopy revealed old blood in the terminal ileum without evidence of active bleeding or mucosal lesions. Despite a negative Meckel’s scan and mesenteric CT angiogram showing no evidence of mesenteric bleeding, her hemoglobin continued to decline to 5.3 on hospital day 20, at which time the patient received two units of PRBCs. Abdominal CT with oral contrast showed mild extravasation in the jejunal loop (Fig. 1a and b). A PE was then performed, which showed blood refluxing from the jejunum distal to the end of the scope, but no source was visualized. Another mesenteric angiogram was performed, which noted GI bleeding and she was transferred to the ICU to be stabilized.

Due to persistent melena and down trending hemoglobin despite multiple transfusions, general surgery was consulted. Upon exam, the patient just had a large bowel movement with melena and multiple blood clots and was complaining of moderate abdominal cramping and diffuse abdominal tenderness to palpation without guarding or peritoneal signs. She was hemodynamically stable after receiving blood products, and the decision was made to perform a diagnostic laparoscopy/laparotomy, hand-assisted PE and possible small bowel resection. Intraoperatively, two bleeding nodules in the jejunum were identified and resected. No intraoperative or postoperative complications were noted, estimated blood loss was 10 ml and the patient was discharged on postoperative day 2. Final pathology identified normal small bowel mucosa with multiple foci of arteriovenous malformations (AVMs) without any evidence of malignancy. On 2-week postoperative follow-up, the patient presented with no signs of infection or postoperative complications and return of normal bowel function. She reported complete resolution of melena and no further syncopal episodes.

Patient 2 was a 57-year-old male with no pertinent medical history who presented with persistent iron deficiency anemia despite prior negative findings on upper endoscopy and colonoscopy. At this time, the patient was hemodynamically stable and without any acute symptoms of anemia, melena or hematochezia. A CE was then performed and identified a distal jejunal mass. An attempt to reach the mass via spiral enteroscopy was unsuccessful, and an India ink tattoo was placed at the most distal location possible. The patient was then taken for a diagnostic laparoscopy/laparotomy, push small bowel enteroscopy and small bowel resection. During the procedure, the jejunal tattoo was identified ~60 cm distal to the ligament of Treitz. Manual advancement of the small bowel directly over the enteroscope identified a pedunculated lesion in the small bowel lumen 40 cm distal to the tattoo. The lesion was then resected and taken for pathology. No intraoperative complications were noted and estimated blood loss was ~10 ml. No postoperative complications were noted, and the patient was discharged on postoperative day 2. The final pathology showed a hamartomatous polyp with smooth muscle core and lobular architecture, which was diagnostic of Peutz-Jeghers syndrome. At the 2-week postoperative follow-up, the patient showed no signs of infection or postoperative complications and had returned to normal bowel function. Final pathology report noted findings consistent with a hamartomatous polyp with no concerns for malignancy.

**Figure 2:**
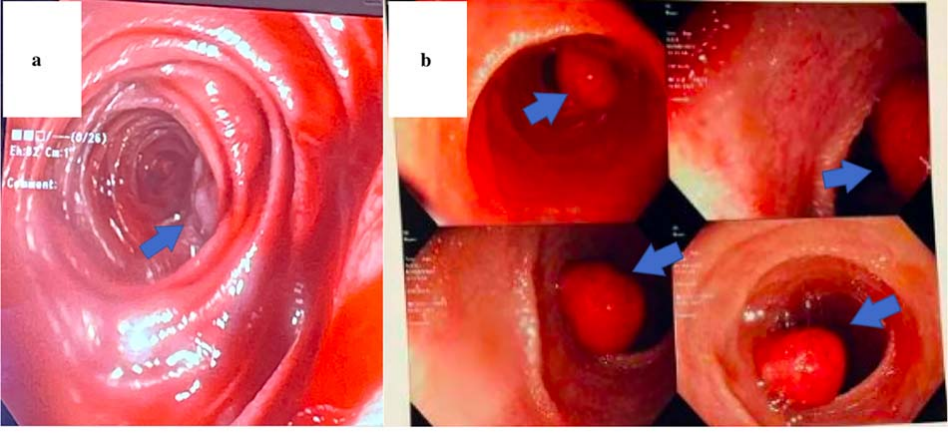
Endoscopic view of AVM identified in Patient 1 (**a**) and hamartomatous polyp in Patient 2 (**b**).

The same surgical approach was used for each patient. After laparoscopic exploration of the abdomen and lysis of adhesions where necessary, a PE and subsequent small bowel resections were performed. An upper midline incision was made, and the bowel was eviscerated. Simultaneously, the gastroenterologist began by advancing the upper endoscope orally to the max distance of scope, ~50–60 cm within the small bowel. The remainder of the small bowel was advanced manually to the ileocecal valve using the scope to visualize the lumen and external mucosa of the bowel in both anterograde and retrograde fashion (Fig. 2a and b).

**Figure 3:**
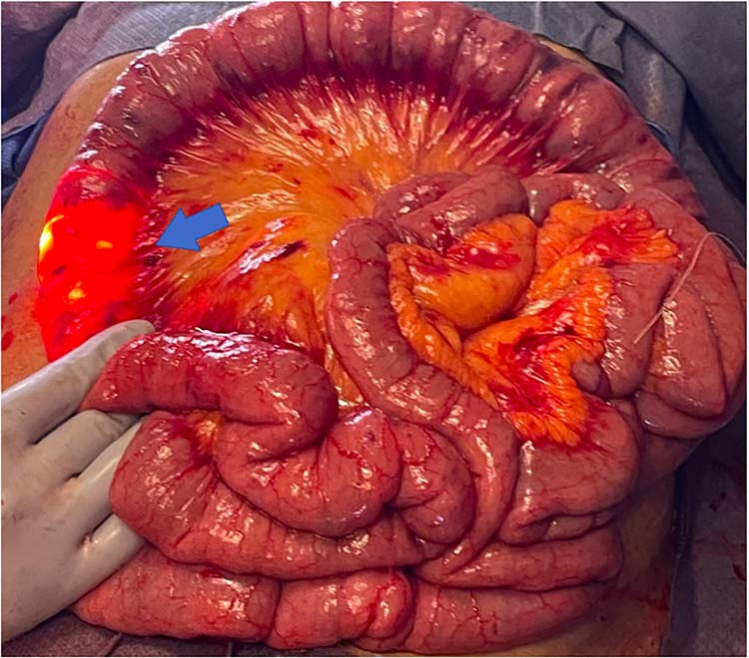
Transillumination of eviscerated small bowel during IOE.

**Figure 4:**
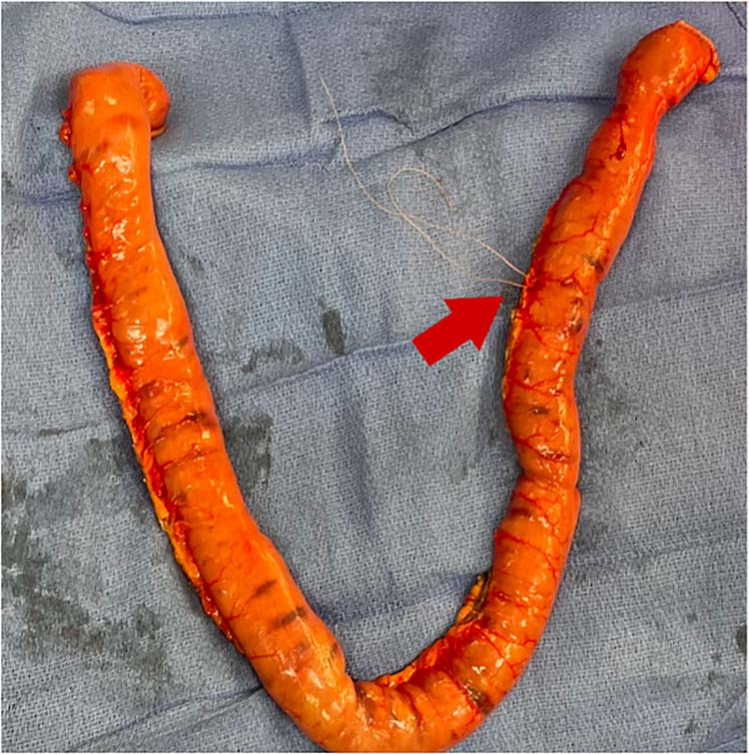
Small bowel resection specimen with 3–0 Vicryl suture marking (red arrow).

During antegrade push and retrograde pullback, dual visualization of the small bowel by the endoscopist intraluminally and by the surgeon externally with the transilluminated bowel was performed (Fig. 3). Once the lesions were identified, the abutting mesentery was marked with 3–0 Vicryl. The scope was continued in a retrograde manner and removed. Small bowel resections were then performed ~5 cm proximal and 5 cm distal to the identified sources marked with 3–0 Vicryl suture (Fig. 4). Defects in the mesentery were created at each end with cautery, and a GIA 75 blue load was fired at each site. Side-to-side functional small bowel anastomosis was performed by excising the antimesenteric portion of the small bowel with Mayo scissors and firing a single GIA 75 load/blue load. Anastomoses were inspected prior to closure to ensure no intraluminal hemorrhage was present. The mesentery was then closed with a running 3–0 Vicryl. Fascial closure was performed with running loop PDS and skin staples.

## DISCUSSION

Evaluation for deep small bowel bleeding requires both a trained endoscopist and the technology available to rapidly identify the ongoing bleed. This can prove difficult for patients who present to rural hospitals, where resources such as CE or spiral enteroscopy might not be readily available. In addition to these barriers, the use of a spiral and PE carries a greater risk of perforation compared with normal endoscopy and is often unable to reach the source of bleeding past the proximal jejunum [4]. On the other hand, CE is a noninvasive method of inspecting the entirety of the small and large intestines. The diagnostic yield of CE in adults with persistent iron deficiency anemia, as seen in Patient 2, has been reported at 66.6% [5]. Among the limitations of CE are the commonly reported poorly visualized portions of the small intestine (duodenum and proximal jejunum) and its use solely as a diagnostic tool, with the inability to sample lesions or perform therapeutic interventions [5, 6]. Estimation based on time and passage of the capsule can also be misinterpreted. Studies evaluated in Kim *et al*. refer to malignancies initially missed on CE, which were later identified on further imaging studies such as CT or MR enterography (CTE or MRE) [5, 7]. With consideration for the limitations of both CE and spiral endoscopy, IOE may be a viable therapeutic intervention, particularly in a rural setting, for a patient presenting with an ongoing deep small bleed.

The diagnostic yield of IOE in small bowel bleeding is reported to be between 56 to 88%, compared with that of VCE and simple PE, 56–66.6% and 25–56%, respectively [5, 8]. The recent retrospective single-center study by Pal *et al*. published data in support of the IOE in the diagnostic workup of small bowel bleeds. The study identified that IOE helped establish a diagnosis in an additional 31% cases with a negative preoperative workup and changed the management in 37.1% patients by locating new or additional lesions. This study identified improved statistics regarding the use of IOE compared with previous studies of its kind, presenting a diagnostic yield of 91.3% in small bowel bleeding [2].

**Figure 5:**
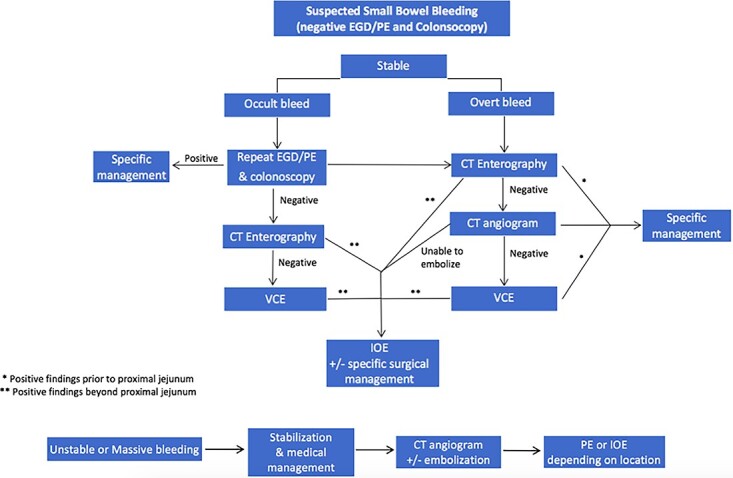
Proposed algorithm for the management of suspected small bowel bleeding in stable and unstable patients.

Four main factors should be evaluated when considering the use of IOE in the management of suspected small bowel bleed: (i) stability of the patient, (ii) occult versus overt bleeding, (iii) resources available, (iv) how will diagnostic test results impact further management [1]. Figure 5 provides a proposed algorithm outlining where IOE should be considered for the treatment or diagnosis of small bowel bleeds or in settings with limited access to deep endoscopy technology or specialized endoscopist. The first step is the evaluation of patient stability. A hemodynamically stable patient presenting with evidence of an ongoing GI bleed must first undergo colonoscopy and upper endoscopy (EGD or push endoscopy). If negative, a CTE is warranted to differentiate between possible etiologies, unless clinicians feel the patient would benefit from a repeat upper and lower endoscopies due to poor bowel preparation or other external factors. If the source remains unidentified, the decision for use of deep small bowel endoscopy versus IOE should be considered. We propose that IOE be heavily considered at this point in rural settings, where specialized endoscopists or diagnostic imaging modalities are not as readily available. The identified etiology and location on CTE or VCE dictate subsequent management. If a mass or significant extravasation from the small bowel is found distal to the proximal jejunum, the next step in management should be IOE. It is vital to utilize this algorithm in conjunction with clinical presentation and diagnostic results. For example, if severe extravasation is identified prior to the jejunum, it might be prudent to attempt arterial embolization prior to IOE if available. Nuclear medicine scans were excluded from this algorithm because they add a great cost and often offer little information to purposefully direct management in most patients. In addition, these modalities are sparse in rural areas.

As an invasive procedure requiring general anesthesia, IOE carries greater risks than the previously mentioned diagnostic methods, but, overall, is a safe procedure that can be performed by a general surgeon and endoscopist. Despite their varying level of acuity and comorbid conditions at presentation, both patients received the same treatment, because they reached a point in the algorithm in which the benefits outweighed the risks of IOE. Understanding the place of intraoperative PE is especially important in the treatment of unstable patients and those presenting in a rural setting where access to deep enteroscopy technology and trained specialists is limited.
